# Emerging anthropogenic circularity science: principles, practices, and challenges

**DOI:** 10.1016/j.isci.2021.102237

**Published:** 2021-02-25

**Authors:** Xianlai Zeng, Jinhui Li

**Affiliations:** 1 State Key Joint Laboratory of Environment Simulation and Pollution Control, School of Environment, Tsinghua University, Beijing 10084, China

**Keywords:** Green Chemistry, Engineering, Energy Sustainability, Green Engineering, Materials Science, Materials Processing

## Abstract

Material depletion over reliance of linear economies and environmental pollution may be resolved by applying the principles and practices of anthropogenic circularity science. Here we systematically review the emergence of anthropogenic circularity science in the interdisciplinary development of green chemistry, supply chain, and industrial ecology at different scales. The first, second, and third laws of circularity chemistry are proposed as forming the basic principles of circularity science. To close the loop on critical materials, these three basic principles have been exemplified in the anthropogenic circularity practices. We highlight the spatial distribution of critical metal, waste generation, and recycling rate. Future opportunities and challenges for a circular economy and urban mining will predominate in anthropogenic circularity. Therefore, anthropogenic circularity science will play an increasing role in enabling a smooth transition to a circular society.

## Graphical abstract

**Figure:**
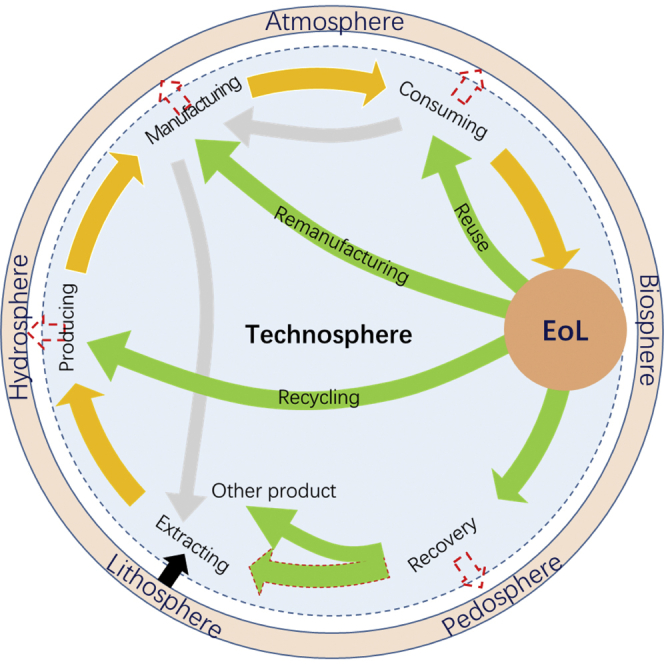

## Introduction

Anthropogenic activity driven by chemistry is leaving a pervasive and persistent signature on Earth so that some material cycles have been significantly modified over the past century (Waters et al., 2016). Global sustainability is declining in an unprecedented rate due to severe resource depletion and serious environmental degradation (IRP, 2017; Liu et al., 2015; OECD, 2019). The United Nations in 2015 set out a transformational agenda for a sustainable 21st century with the adoption of 17 Sustainable Development Goals (SDGs) to protect the earth and its inhabitants. For example, SDG 12 states as follows: sustainable consumption and production focuses on improved utilization of our over-stressed and critical materials, doing more with less, and adoption of circular rather than linear economies. This is very important as increasing consumer demands from an ascending population with the rising aspirations for a better future have led to concerns over the security of supply and accessibility of many elements within the Periodic Table that are consumed in chemical processes and manufacturing. Currently, many processes for generating chemical products are not sustainable owing to many negative impacts for resources and the environment (Moreau et al., 2019; Poliakoff and Licence, 2007).

One of humankind's biggest challenges over the 21st century is how to provide adequate resources for civilization. Most geological materials extracted so far have been transformed into products and finally to waste, i.e., a linear economy. As a result, global human-made mass has exceeded all living biomass (Elhacham et al., 2020). Geochemistry plays a pivotal role, from the processes that accumulate elements into ore bodies, to developing exploration techniques that are used to find them (Sverdrup and Ragnarsdóttir, 2014). A circular economy will put much concern on chemistry to make the innovative products, using the renewable feedstock in an eco-design manner. Furthermore, the substances these products are made from will increasingly be handled as a secondary resource and not simply disposed of as waste (Clark et al., 2016). A circular economy is recognized as an effective approach to alleviate and even solve global issues, and chemical processes are a fundamental part of this. We provide a first analysis of the principles, practice, and challenges of the emerging anthropogenic circularity science.

## Emergence of anthropogenic circularity science

Circularity of material is an emerging word in recent year, whereas the circular economy is tentatively employed to solve societal and ecological problems (Anastas, 2020). It is defined as a closed loop (Saidani et al., 2019), in particular through waste recycling or circular economy (Billiet and Trenor, 2020; Cobo et al., 2019; Tercero Espinoza et al., 2020). Anthropogenic circularity science seeks to create a closed-loop material flow typically linking the green chemistry, supply chain, and industrial ecology at different scales (Cooper et al., 2020; Heisel and Rau-Oberhuber, 2020; Linder et al., 2017).

### Green chemistry

On the microscopic scale, green chemistry is regarded as the design of output and processes that eliminate the use and generation of unfriendly substances. It is derived from the growing demand for more sustainable processes in the chemical world, devoting to reduce or avoid the utilization of hazardous substances and to minimize wastes generated from the chemical reactions (Anastas and Lankey, 2000). Some mature chemical processes, which are often based on technology developed in the first half of the 20th century, may no longer be welcome owing to today's environmental awareness. “Eco-economics” has become the driving force for new products and processes, whereas the cost of waste disposal and pollution control is increasing (Clark, 1999).

The most important aspect of green chemistry is the philosophy of design. A dozen principles of green chemistry have been identified as ‘‘design rules’’ to help producer reach the sustainability (Anastas and Eghbali, 2010; Duan et al., 2015). From the environmental perspective, the pursuit of waste minimization has elevated green chemistry (Beach et al., 2009). The adoption and development of green chemistry has been catalyzed by the formulation of principles and metrics that would guide the design of sustainable chemicals (Mulvihill et al., 2011). Although this field has been developed rapidly in last 25 years, it is still in the infant stage. There is no doubt that the development and implementation of green chemistry will contribute strikingly to the sustainable development of modern society (Song and Han, 2015).

### Closed-loop supply chain

On the mesoscopic scale, the supply chain is a system of organizations, people, activities, information, and resources for the flow of product or service from supplier to customer. Traditionally, supply chain activities involve the transformation of natural resources, raw materials, and components into finished products that are delivered to the end consumer.

**Figure 1:**
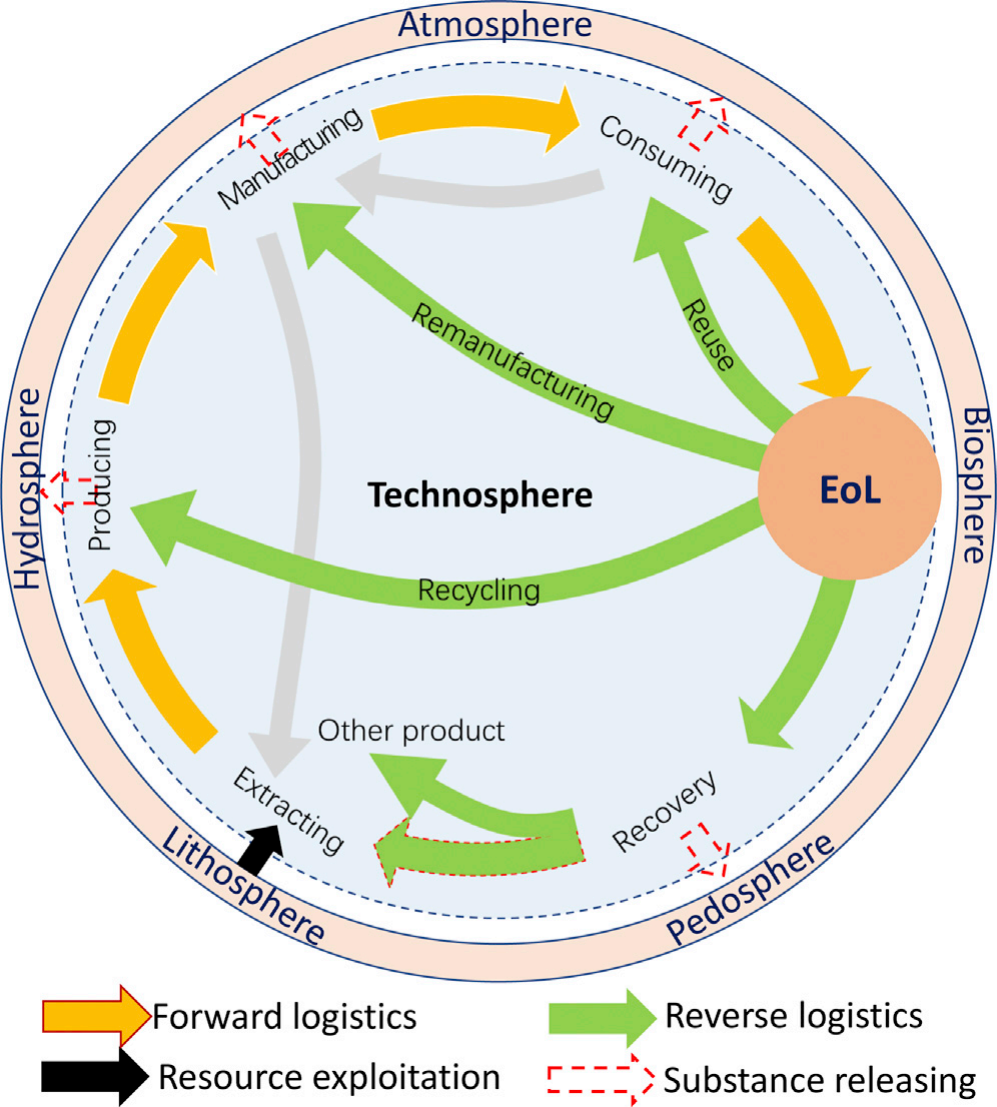
Systematic material cycling diagram for closed-loop supply chain Note: yellow arrow indicates clockwise supply chain, and green arrow and gray arrow indicate count-clockwise supply chain. Adapted with permission from (Zeng and Li, 2018), copyright of Science China Press (2018).

Currently, supply chain is divided into forward and reverse supply chain, which could constitute closed-loop supply chains (O'Rourke, 2014). Forward supply chain covers natural resource extraction, manufacturing, transportation, retail operations, product consuming, and end-of-life (EoL) disposal. Chemistry is the key driver mainly in extracting and producing to enable the function of product. Consumers can take advantage of the product after physical distribution. The process can be out-looked as forward logistics of products. Waste is generated during the processes of manufacturing (called as new scrap) and consuming or after the product has been used for long periods and approaches the EoL phase as old scrap. All the processes of product are composed of forward supply chains and thus, a linear economy (Figure 1).

In reverse supply chain, some high-quality old scrap can be reused as product; common old scrap can be disassembled for remanufacturing, and the remanufactured products are typically upgraded to the equal quality standards of new products that can be resold in markets (Savaskan et al., 2004); some can be dismantled for recycling and entered to original production processes; and also the residues can be recovered as raw materials using green chemistry (Figure 1) (Li et al., 2015b).

### Industrial ecology

On the macroscopic scale, the concept of industrial ecology is referred to natural ecological systems. In nature, an ecological system runs through a food chain or web of connections in which organisms live and consume each other and each other's waste (Allenby, 2006). Similar thinking can be grafted into industrial ecosystems (Graedel, 2000). In the early stage, unlimited resources were utilized in industrial process and generated unlimited waste. Currently, when energy and limited resources are utilized in complex chemical industry within recycling, limited waste can be discharged.

The definition of industrial ecology has evolved since 1980s (Clift and Druckman, 2016). Nowadays, industrial ecology conceptualizes industry as an emerging discipline that operates in a similar way as natural ecosystems, where the waste or by-product from one process is used as an input into another one (Ayres and Ayres, 2002). Industrial ecology is original from natural ecosystems and attempts to transfer from a linear to cyclical or closed loop system. Like natural ecosystems, industrial ecology is in a continual state of flux. A basic target of industrial ecology is to design or modify an industrial system such that the wastes of one industry can serve as the feedstocks for another industry (Lifset, 2014). Therefore, the insight of industrial ecology no longer focuses solely on technology and has covered the nexus of sustainability through multidisciplinary partnerships (Bunker, 1996; D'Odorico et al., 2018; Liu et al., 2018a).

The conceptual framework of industrial ecology is defined as transforming the use of materials in product chain from a linear to a circular model, which is used to uncover the interactions of the high-technological society with the environment (Harper and Graedel, 2004). Essentially, industrial ecology heralds a call for the operation of closed-loop supply chain from manufacturing, consumption, and recycling, to disposal (Linton and Yeomans, 2004). Nowadays, growing materials ecology is about the evolution of a new generation of products that can be easily recycled elsewhere at the end of their life (Allwood, 2016). A modern vehicle, for instance, is a very complicated product capsuling many thousands of parts and components. After the obsolescence, the vehicle is dismantled and most of them are recycled, especially for the metals and plastics (Collier and Alles, 2010; Ohno et al., 2017).

## Three laws of anthropogenic circularity chemistry

**Figure 2:**
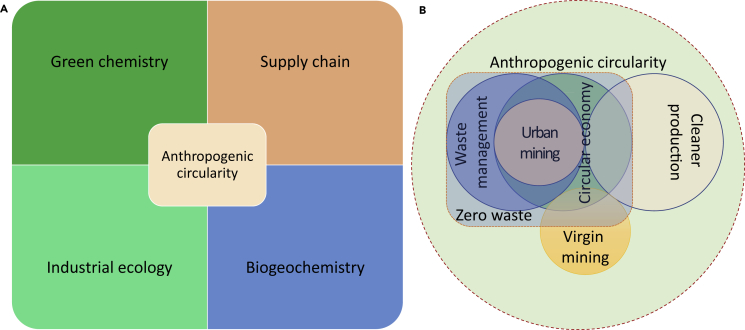
The defined boundary of anthropogenic circularity science (A) Disciplines; (B) practices.

The development of many-scale disciplines in terms of green chemistry, closed-loop supply chain theory, and industrial ecology are boosting the emergence and evolution of anthropogenic circularity science (Figure 2A). Circularity chemistry is defined as the combination of biogeochemical cycle and anthropogenic recycling, which is the fundamental rule to uncover the circular economy for the sustainable development. Three principles are expressed as first, second, and third laws of circularity chemistry from the macroscopic and mesoscopic scales to the microscopic scale.

### First law of circularity chemistry

The first law of circularity chemistry (Equation 1), also known as the fundamentals of constant abundance, states that the relative contents (or abundance) of various moved chemical elements in the universe are constant (Jin, 2014).

(Equation 1) $$F=\frac{Ma}{\sum Mn}$$

where *F* is the abundance of elements (%), *Ma* is the content of element (tons, 1 t = 10^3^ kg), and *Mn* is the total sum of all the elements' content in tons.

One of the persistent problems of interest to environmental and allied scientists concerned with the chemistry of meteorites and planets has been the original composition of the solar system. When the abundance of elements is constant, the total mass of all elements in the universe will be constant.

The first law of circularity chemistry indicates the importance and scope of anthropogenic circularity. The Earth's natural resources are limited, and material supply from urban mining is much needed to meet the shortage of virgin resources (Ciacci et al., 2016). The scope of circularity chemistry covers the 83 types of natural elements. With circularity chemistry, any waste containing any of the 83 elements is considered as a raw material to recycle for new products.

### Second law of circularity chemistry

The second law of circularity chemistry is the principle of material cycling, which is a universal law of ecological systems. It means that all the elements and products made from them are moving along cyclical routes in an ecological systems (Jin, 2014).

#### Composition and structure of the ecosystem

Driven by the biological, physical, and chemical components, the ecosystem consists of two main components, i.e. community, which is called biocoenosis and habitat (biotope). Functionally, the two components of the ecosystem (autotrophs and heterotrophs) can be recognized usually with four constituents, i.e. abiotic, producers, consumers, and decomposers (Liwarska-Bizukojc et al., 2009). The four constituents play different roles. For example, producers (e.g., plants and algae) acquire nutrients from inorganic sources that are supplied primarily by decomposers, whereas decomposers, mostly fungi and bacteria, acquire some elements from organic sources that are supplied primarily by producers (Naeem et al., 2000).

#### Material cycle

According to the scope, the biogeochemical cycles consist of a geochemical and biological cycle. The geochemical cycle is the pathway of compounds and elements, covering the adsorption in biont (a discrete living organism that has a specified mode of living), back to the environment via dying, residue, or excrement of biont, and further utilization for biont through five spheres, i.e. atmosphere, hydrosphere, pedosphere, lithosphere, and biosphere. The geochemical cycle supplies the external environment for the biological cycle. The biological cycle is the pathway for elements via adsorption, further utilization, decomposition, and new adsorption in ecosystem.

A material cycle is characterized by its indestructibility and circularity. During transformation, material and energy can be changed in form but can never be destroyed. But the material cycle differs from energy flow because energy transfer is irreversibly leaving the ecosystem, whereas material can be recycled. Material is limited and heterogeneous in distribution and can be recycled frequently owing to its perpetual utilization in the ecosystem.

The second law of circularity chemistry uncovers the direction from the environment (e.g. atmosphere), producer (e.g. green plant), consumer (e.g. animal, human), and decomposer (e.g. microorganism). Therefore, this law is also known as the law of conservation of mass. Material can be changed in form but can never vanish during the cycle. The second law of circularity chemistry indicates the feasibility of recycling.

### Third law of circularity chemistry

The third law of circularity chemistry (Equation 2) is the principle of zero waste that chemical reactions should be designed to tentatively achieve zero emission of waste.

(Equation 2) ∑W→0

where *W* is the amount of waste.

The third law can also be explained via the principle of atom economy or atom efficiency, which is the conversion efficiency of a chemical process in terms of all atoms involved and the desired products produced (Equation 3). Atom economy is an important concept of green chemistry and a widely used metric for measuring the "greenness" of a process or synthesis (Trost, 1995). However, it must be noted that not all 100% atom efficient processes can automatically be considered to be green.

(Equation 3) $$E=\frac{D}{T}\times 100\%$$

where *E* is the atom economy or efficiency, *D* is the molecular mass of desired product, and *T* is the molecular mass of all reactants.

Ideally, a manufacturing process would design some related reactions so that all atoms are included in the product structure. By the conservation of mass, the total molecular mass of the reactants is the same as the total molecular mass of the products. In an ideal chemical process, the amount of starting materials or reactants equals the amount of all desired products generated with no atom wasted.

The third law of circularity chemistry in the microscopic scale implies the importance of maintaining an ecological balance. The natural material cycle is always a process with nearly zero waste emission. If, this process is broken and/or ecological balance is destroyed, then waste will be inevitably generated that may endanger human life or the health of the environment.

## Practices of anthropogenic circularity science

Based on the principles of circularity chemistry, zero waste, circular economy, and urban mining are the most relevant practices from macroscopic to microscopic level to maintain the anthropogenic circularity (Figure 2B).

### Zero-waste city and society

The term “zero waste” was first proposed by Dr Paul Palmer in 1973 for recycling materials from chemical product (Palmer, 2005). Formal waste management has progressed from open dumping and uncontrolled landfilling to composting, recycling, waste-to-energy, and controlled landfilling process (Liu et al., 2020). In a zero-waste system, material flow is circular, supported by the second law of circularity chemistry. Thus, the same materials are used many times until the optimum level of consumption is achieved. Within the system the discarded products could be reused, repaired, sold, or redistributed. If reuse or repairs are not economical, then they, as the above analysis of reverse logistics, can be recycled or recovered from the waste stream and used as inputs, substituting the demand for virgin resources and the associated mining costs.

**Figure 3:**
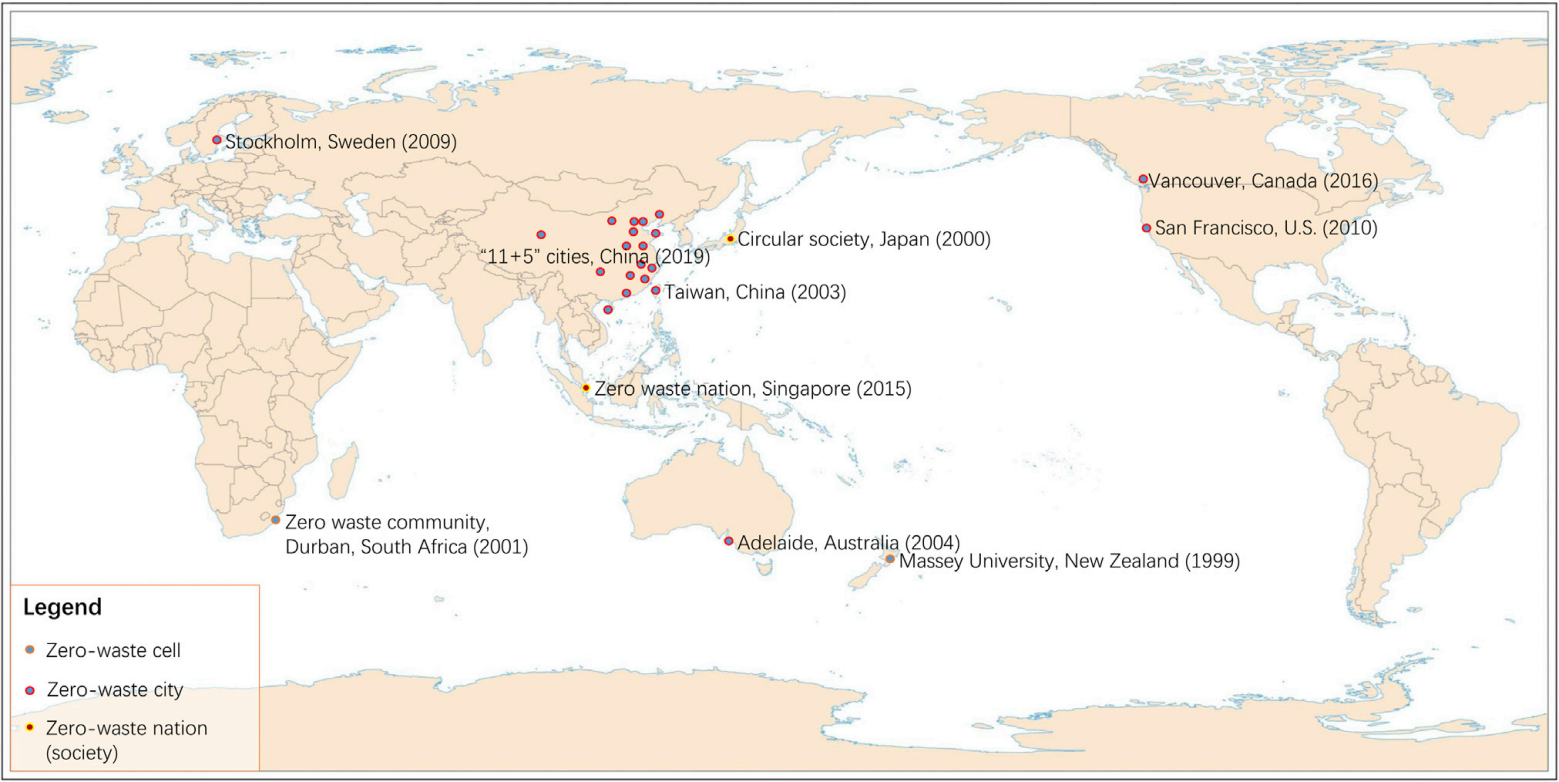
Main practices of zero waste program in the world Data source from Curran and Williams, 2012; Lang, 2005; Matete and Trois, 2008; Rathoure, 2019; Young et al., 2009).

Zero waste represents a shift from the traditional industrial models in which wastes are considered as the norm, to integrated systems in which everything has its use. In practice, some programs of zero waste have been implemented in numerous communities, cities, and countries (Figure 3). New Zealand and Japan were the pioneers of zero waste dream from community to society. In 2000, Japan initiated the circular society with the focus on waste management and material depletion (Geng et al., 2013). On December 29, 2018 China State Council released one ambitious zero-waste city program. The initial pilots were set for 11 cities and 5 regions (named as “11 + 5”), covering Shenzhen, Baotou, Tongling, Weihai, Chongqing, Shaoxing, Sanya, Xuchang, Xuzhou, Panjin, and Xining, as well as Xiongan, Beijing E-town, Tianjin Eco-city, Guangze, and Ruijin (Li and Zhuo, 2019). The obtained successful experience and model would be hopefully extended to other Chinese cities. Through maximizing reduction and recycling and minimizing the landfill and environmental impact, this philosophy is devoted to hopefully push the urban green development pattern based on the innovation, coordination, green, open, and share.

### Circular economy

**Figure 4:**
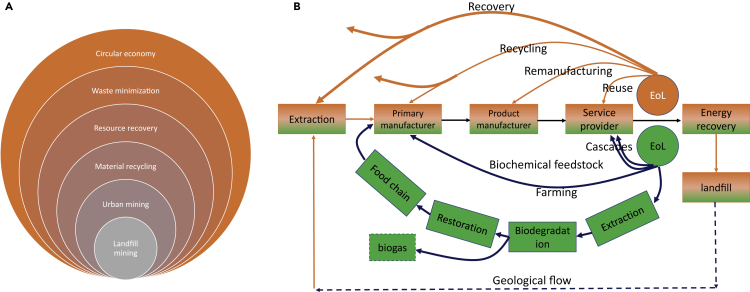
Circular economy (A) Boundary; (B) scope. Note: blue arrow and orange arrow indicate biosphere and technosphere, respectively.

As noted earlier, the essence of the second principle of green chemistry and SDG 12 is designed for material efficiency. This is stimulating the much-needed move from a traditional linear flow of materials in an “extract-manufacture-use-dispose” economy, to a greener, circular economy that devotes to minimize waste through deliberate design of products, processes with resource consumption, and recycling at the outset (Sheldon, 2016). Circular economy employs the industrial ecology law to realize the special goal of resource efficiency and recycling rate. It has evolved from *The Economics of the Coming Spaceship Earth* in 1966, and *Economics and the Environment*: *A Materials Balance Approach* in 1970s, to a theory of circular economy promoted by Ellen MacArthur Foundation in 2010s (EEA, 2016). Its boundary has surpassed landfill mining, urban mining, material recycling, material recovery, and waste minimization (Figure 4A) (Cossu and Williams, 2015).

The drive to shift material composition of consumables from technical activity toward biological materials and to have those cascades through different applications before extracting valuable feedstock and finally re-introducing their materials into the biosphere encompasses the core principles of a restorative circular economy (Jackson et al., 2014). Therefore, the circular economy addresses an industrial economy in which material flows keep circulating at a high rate without the material entering the biosphere unless the biological nutrients (Figure 4B) (Lehmann et al., 2014). In the technosphere, recycling has now become an integral component of the supply chain. Recycling of used products and the related logistics management provide a significant opportunity to circular economical industries (Liu et al., 2018b; Mishra et al., 2011). Nowadays, circular economy is booming not only in industrial nations (e.g., the EU, Japan) but also in rapidly emerging and high growth countries (e.g., China) (Geng et al., 2013; Manhart et al., 2014).

Regulated by the second law of circularity chemistry, the technical process of circular economy is emerging. When the product reaches the EoL phase, the emerging technical hierarchy of circular economy can be obtained: high-quality spent product can be reused again for consumption; broken product can be disassembled to get the components for remanufacturing to re-enter the consumption chain; waste product can be dismantled for recycling (e.g. mechanical crushing, screening) to implement original production (closed loop) or sent to another production process for new cycle (open loop); other waste after recycling can be recovered as raw materials though the third law of circularity chemistry (closed loop) or put into another production process for other new cycle (open loop) (Horton et al., 2018); and any residues are disposed of a controlled landfill plant (Figure 4B) (The-Ellen-MacArthur-Foundation, 2012).

### Urban mining

It is the process to reclaim the compounds and materials from old scrap, providing a systematic and goal-oriented management of anthropogenic material stocks and waste (products and buildings), considering long-term environmental protection, resource conservation, and economic benefits (Cossu, 2013; Klinglmair and Fellner, 2010). Urban mining of electronic and electrical waste (e-waste) is becoming more economical than virgin mining of natural mineral (Zeng et al., 2018). Therefore, it is becoming an important solution to achieve the win–win objectives of enhanced resource sustainability and improved environmental quality, while developing a robust circular economy based upon reuse and recycling (Krook and Baas, 2013).

In light of the first law of circularity chemistry (Equation 1), an anthropogenic mine (or called secondary resource) mainly covers e-waste, EoL vehicles, and waste cable and wiring, representing a huge amount of valuable and precious materials (Wang et al., 2020; Zeng et al., 2020). The potential of urban mining also covers the quantities of materials that are buried in landfills and are hibernating in cities, which if mined could dramatically reduce the amounts of virgin materials (Jones et al., 2013). Within the second law of circularity chemistry (Equation 2), urban mining illustrates resource flow direction from stock in anthropogenic mine to utilization in production and consumption (Figures 1 and 4). The major approaches involve remanufacturing, recycling, and recovery. Therefore, urban mining can maintain society to alleviate the pressure on natural resources depletion and meanwhile to decrease air and water pollution from the effluents of the landfills.

The third law of circularity chemistry (Equation 3) has been extensively employed in urban mining process. A first disassembly or dismantling or de-manufacturing is generally dispensable because many types of anthropogenic mines are mainly composed of physically combined structures (Ruan and Xu, 2016; Sun et al., 2016). In the light of elemental metals in anthropogenic mines, mechanical/physical technologies are identified to separate metals from nonmetallic components (Xu et al., 2019). A pyrometallurgical process is often adopted for metal extraction, although the expensive equipment required has limited its use in medium- and small-sized enterprises. Hydrometallurgical process is relatively effective and easy to operate, but with high selectivity of targeted metals toward an eminent recovery efficiency (van Hullebusch et al., 2019; Wang et al., 2017). Using lithium-ion batteries as a typical example, a short-cut recycling of cobalt and lithium was directly achieved using oxalic acid, which afforded dissolution and precipitation of lithium oxalate salts and complexes (Zeng et al., 2015).

## Anthropogenic circularity performance of waste and metal

In larger-scale earth science, a geochemical cycle is a pathway by which a chemical substance/element moves through both biotic (biosphere) and abiotic (lithosphere, atmosphere, and hydrosphere) components of the Earth (Plane et al., 2015). Anthropogenic activities have significantly altered the global cycles of metals in Earth's ecosystems (Ellis, 2011). Massive amounts of metals have been extracted from the lithosphere to be used in the flourishing electronics and new and renewable energy industries (Chancerel et al., 2015; Christian et al., 2014). Metals are not “running out” or being destroyed but rather are being dispersed throughout the technosphere, making recapture both highly problematic and often very costly (McLellan et al., 2016; Tilton, 2015). There is a growing need to develop the best approach to recycle them from waste repositories, current or historic, both for hazard avoidance and potentially, as a new source of elements for industry (Reuter et al., 2019).

**Figure 5:**
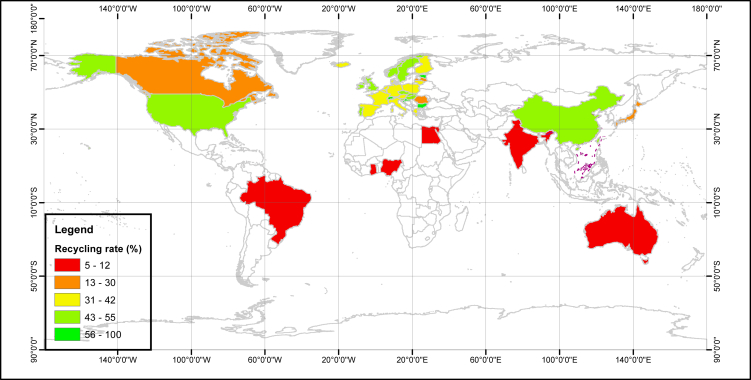
E-waste recycling rate in the world Note: data source from SM Table S1.

Hunting treasure from e-waste, typically as high environmental-risk and material-stock waste, has absorbed wide concern since 2000 (Ogunseitan et al., 2009). Some developed countries tended to shirk responsibility to dump their e-waste in developing countries (Wang et al., 2016). Many developing countries like China have struggled strenuously to eliminate the informal recycling and illegal importation for two decades. Currently, some European countries, China, and the US are leading in the formal recycling rate (Figure 5). The global average was only around 20% (Li et al., 2015a). The recycling rate of most of the countries and regions is still less than 10% so that we are still far away from a closed-loop system (Reck and Graedel, 2012). It indicates a huge potential of anthropogenic circularity in the near future.

## Opportunities, challenges, and prospects

Innovation through green chemistry and chemical technologies combined with sustainable manufacturing practices will enforce. The United Nations Sustainable Development Summit (25^th^ September 2015) adopted the document, entitled “Transforming Our World: the 2030 Agenda for Sustainable Development,” setting out 17 SDGs for removal of poverty, protection of the planet, and ensuring prosperity for all (UNGA, 2015). In particular, SDG 12 strives to ensure sustainable consumption and production patterns; promotes material and energy efficiency and sustainable infrastructure; and provides access to basic services, green, and decent jobs as well as a better quality of life for all. Key targets of SDG 12 including the substantial reduction of waste generation and the notion of a systemic approach and cooperation among actors operating in the supply chain will oblige a huge room for cyclical chemical.

### Future opportunities

The concepts of “Zero Waste” have been implemented in a couple of countries, cities, as well as in a range of companies (Curran and Williams, 2012; Lang, 2005; Matete and Trois, 2008). On December 29, 2018 China State Council released the solution to establish zero-waste city pilot. Through maximizing reduction and recycling and minimizing the landfill and environmental impact, this philosophy is devoted to hopefully push the urban green development pattern based on the innovation, coordination, green, open, and share.

Three scales of cycling, small cycling, moderate cycling, and large cycling are occurring in various spheres of the earth. In the biosphere, the natural ecosystem is driven by natural energy flow and human needs. In the technosphere, typical activities such as urban mining, zero waste, and circular economy are driven mainly by human needs. The anthroposphere is partly consisted of the biosphere and the technosphere. However, in all spheres, the global ecosystem is driven by geodynamic and human needs (Table 1).

**Table 1:** Summary for different cycling in various spheres of the earth

Sphere	Typical activity	Main process	Cycling scale	Driver
Biosphere	Natural ecosystem	Cascades	Small cycling	Energy flow
		Farming	Small cycling	Social needs
		Food chain	Moderate cycling	Energy flow and social needs
Technosphere	Urban mining Zero waste Circular economy Industrial ecosystem	Reuse	Small cycling	Social needs
		Remanufacturing	Small cycling	Social needs
		Recycling	Moderate cycling	Social needs
		Recovery	Moderate cycling	Social needs
All spheres	Global ecosystem	Geochemical cycling	Large cycling	Geodynamics and social needs

The circular economy provides a huge opportunity to improve material efficiency and environmental protection. Industrial symbiosis and circularity chemistry offer many possibilities for closed loop rather than linear materials flow, especially for poorly recovered critical elements. The current extraction of natural minerals is not sustainable in terms of the consumption of many elements in industrialized countries owing to their geologic scarcity and spatial shortage (Henckens et al., 2014; Klimek et al., 2015; Lusty and Gunn, 2015). On the other hand, waste production will peak in this century when population growth and urbanization will outpace waste reduction (Hoornweg et al., 2013). Many resources are considered to be close to “peaking” including lithium, helium, copper, and the rare earth elements (Rustad, 2012).

A sustainable material industry will be based on a closed loop of material, which is as far as possible free of quantitative and qualitative losses in the technosphere. But as a global society, we are currently far away from a closed-loop material system (Reck and Graedel, 2012). Urban mining is going to be increasingly important to relieve the coming resource crisis (Ayres and Peiro, 2013; Gauffin et al., 2016; Wen et al., 2015). However, there is still much room for increased efficiency in urban mining, which also offers the valuable opportunity for material recycling worldwide and not simply where the minerals occur naturally. More mining is unavoidable, but increased recycling, substitution, and careful design of new high-technology devices will help meet the growing demand (Vidal et al., 2013).

### Potential challenges and studies

Anthropogenic material cycles are having a huge impact on the chemistry of the planet, leading to the Anthropocene Epoch (Donahue, 2015; Zalasiewicz et al., 2011). Not only geological ores in global metallic mining are becoming scarce but also their grades of the remaining ores are decreasing significantly (Calvo et al., 2016). The above analysis also indicates that anthropological activities have sped up the natural geochemical cycle. However, on at least two scales, the material cycle has declined in the technosphere. At a small scale, any materials are still stored in waste without recycling due to low rates of collection. Meanwhile, a great deal of loss and dissipation of materials have occurred owing to low recycling and a great reliance on landfill (Izatt et al., 2014; Rauch and Graedel, 2007). On an industrial scale, waste or residue released from one enterprise has rarely gone to another enterprise for further production. Moreover, the balance and compatibility of materials, water, and energy yet remain at the developing phase.

Therefore, in the life cycle perspective of material flow, many potential studies still remain to show that more materials can stay with a long retention time at the material in product stage. Because the geological reserve is dynamic, more mineral resources can be discovered at the natural mining stage. Extensive mineral exploration will be required to meet this future resource demand, because many of the undiscovered deposits will be harder to find and more expensive to mine than near-surface deposits located in more accessible areas (Meinert et al., 2016). It provides the opportunities for green mining for material supply using circularity chemistry. Conventional mining process should be improved for cleaner production.

During the manufacturing process, the third law of circularity chemistry should be strictly pursued to reduce the generation of by-product and scrap, including for the organic chemical industry. Catalysis should be expanded and updated to play a bigger role in manufacturing processes (Sheldon, 2016). In addition, material substitutes can also be preferentially considered to choose the materials with low supply risk and low toxicity (Köhler, 2013; Komeijani et al., 2016).

At the EoL stage or urban mining, for instance, e-waste could be a future source of critical material (Williams, 2011); however, the reduction of dissipation of critical metals should have much higher priority (Ylä-Mella and Pongrácz, 2016). Previous practices on urban mining, zero waste, and circular economy are calling for green engineering using circularity chemistry (O'Connor et al., 2016). The basic nature of an urban mine should be clarified, and the clear difference and boundaries between urban mines, natural minerals, and municipal solid waste should be drawn. Then, to achieve metal sustainability, green processes involving pre-treatment, mechanical treatment, hydrometallurgical, or pyrometallurgical process should be developed.

## Conclusions for science and policy

We believe that circularity chemistry should be regarded as a fundamental principle to solve the major issues of resource deletion and environmental pollution. The development of green chemistry, closed-loop supply chains, and industrial ecology has resulted in the emergence of anthropogenic circularity science. The first, second, and third laws of circularity chemistry are proposed as forming the basic principles of anthropogenic circularity. All the three principals have been adopted into some important practices, especially for urban mining for metal recovery, zero waste for circular management, and the circular economy for material efficiency. All anthropogenic practices designed for serving human-kind have accelerated the flow of elements in natural geochemical cycling from the lithosphere, pedosphere, hydrosphere, and atmosphere, to the biosphere and technosphere. However, previous attempts from 1966 to 2018 to develop a form of circular economy approach have revealed many potential challenges. There is clearly a need for more studies on the effective application of anthropogenic circularity science as a part of a circular economy as well as in urban mining. We see anthropogenic circularity science playing an increasingly important role in enabling a transition toward a more sustainable society which can support the world's population without destroying the environment.

### Resource availability

#### Lead contact

Further information and requests should be directed to and will be fulfilled by the lead contact, Xianlai Zeng (xlzeng@tsinghua.edu.cn).

### Materials availability

This study did not generate new unique reagents.

#### Data and code availability

All data are available by contacting the lead author. The study did not generate unique code.

## References

[bib1] Allenby B. (2006). The ontologies of industrial ecology. Prog. Ind. Ecol..

[bib2] Allwood J.M. (2016). Sustainable materials. Nat. Rev. Mater..

[bib3] Anastas P., Eghbali N. (2010). Green chemistry: principles and practice. Chem. Soc. Rev..

[bib4] Anastas P.T. (2020). Circularity. What’s the problem?. ACS Sustain.Chem. Eng..

[bib5] Anastas P.T., Lankey R.L. (2000). Life cycle assessment and green chemistry: the yin and yang of industrial ecology. Green. Chem..

[bib6] Ayres R.U., Ayres L. (2002). A Handbook of Industrial Ecology.

[bib7] Ayres R.U., Peiro L.T. (2013). Material efficiency: rare and critical metals. Phil. Trans. R. Soc. A.

[bib8] Beach E.S., Cui Z., Anastas P.T. (2009). Green Chemistry: a design framework for sustainability. Energy Environ. Sci..

[bib9] Billiet S., Trenor S.R. (2020). 100th anniversary of macromolecular science viewpoint: needs for plastics packaging circularity. ACS Macro Lett..

[bib10] Bunker S.G. (1996). Raw material and the global economy: oversights and distortions in industrial ecology. Soc. Nat. Resour..

[bib11] Calvo G., Mudd G., Valero A., Valero A. (2016). Decreasing ore grades in global metallic mining: a theoretical issue or a global reality?. Resources.

[bib12] Chancerel P., Marwede M., Nissen N.F., Lang K.-D. (2015). Estimating the quantities of critical metals embedded in ICT and consumer equipment. Resour. Conserv. Recycl..

[bib13] Christian B., Romanov A., Romanova I., Turbini L. (2014). Elemental compositions of over 80 cell phones. J. Electron.Mater..

[bib14] Ciacci L., Nuss P., Reck B., Werner T., Graedel T. (2016). Metal criticality determination for Australia, the US, and the planet—comparing 2008 and 2012 results. Resources.

[bib15] Clark J.H. (1999). Green chemistry: challenges and opportunities. Green. Chem..

[bib16] Clark J.H., Farmer T.J., Herrero-Davila L., Sherwood J. (2016). Circular economy design considerations for research and process development in the chemical sciences. Green. Chem..

[bib17] Clift R., Druckman A. (2016). Taking Stock of Industrial Ecology.

[bib18] Cobo S., Levis J.W., Dominguez-Ramos A., Irabien A. (2019). Economics of enhancing nutrient circularity in an organic waste valorization system. Environ. Sci. Tech..

[bib19] Collier P., Alles C.M. (2010). Materials ecology: an industrial perspective. Science.

[bib20] Cooper D.R., Ryan N.A., Syndergaard K., Zhu Y. (2020). The potential for material circularity and independence in the U.S. steel sector. J. Ind. Eco..

[bib21] Cossu R. (2013). The urban mining concept. Waste Manage.

[bib22] Cossu R., Williams I.D. (2015). Urban mining: concepts, terminology, challenges. Waste Manage.

[bib23] Curran T., Williams I.D. (2012). A zero waste vision for industrial networks in Europe. J. Hazard. Mat..

[bib24] D'Odorico P., Davis K.F., Rosa L., Carr J.A., Chiarelli D., Dell'Angelo J., Gephart J., MacDonald G.K., Seekell D.A., Suweis S. (2018). The global food-energy-water nexus. Rev. Geophys..

[bib25] Donahue C.J. (2015). The anthroposphere, material flow analysis, and chemical education. J. Chem. Educ..

[bib26] Duan H., Wang D., Li Y. (2015). Green chemistry for nanoparticle synthesis. Chem. Soc. Rev..

[bib27] EEA (2016). Circular Economy in Europe— Developing the Knowledge Base, Vol EEA Report No 2/2016.

[bib28] Elhacham E., Ben-Uri L., Grozovski J., Bar-On Y.M., Milo R. (2020). Global human-made mass exceeds all living biomass. Nature.

[bib29] Ellis E.C. (2011). Anthropogenic transformation of the terrestrial biosphere. Philos. Trans. R. Soc. A.

[bib30] Gauffin A., Andersson N., Storm P., Tilliander A., Jönsson P. (2016). The global societal steel scrap reserves and amounts of losses. Resources.

[bib31] Geng Y., Sarkis J., Ulgiati S., Zhang P. (2013). Measuring China's circular economy. Science.

[bib32] Graedel T.E. (2000). The evolution of industrial ecology. Environ. Sci. Technol..

[bib33] Harper E.M., Graedel T.E. (2004). Industrial ecology: a teenager’s progress. Technol. Soc..

[bib34] Heisel F., Rau-Oberhuber S. (2020). Calculation and evaluation of circularity indicators for the built environment using the case studies of UMAR and Madaster. J. Clean. Prod..

[bib35] Henckens M.L.C.M., Driessen P.P.J., Worrell E. (2014). Metal scarcity and sustainability, analyzing the necessity to reduce the extraction of scarce metals. Resour. Conserv. Recycl..

[bib36] Hoornweg D., Bhada-Tata P., Kennedy C. (2013). Waste production must peak this century. Nature.

[bib37] Horton P., Allwood J., Cassell P., Edwards C., Tautscher A. (2018). Material demand reduction and closed-loop recycling automotive aluminium. MRS Adv..

[bib38] Bringezu S., Ramaswami A., Schandl H., O’Brien M., Pelton R., Acquatella J., Ayuk E.T., Chiu A.S.F., Flanegin R., Fry J., IRP (2017). Assessing global resource use: A systems approach to resource efficiency and pollution reduction. A Report of the International Resource Pane.

[bib39] Izatt R.M., Izatt S.R., Bruening R.L., Izatt N.E., Moyer B.A. (2014). Challenges to achievement of metal sustainability in our high-tech society. Chem. Soc. Rev..

[bib40] Jackson M., Lederwasch A., Giurco D. (2014). Transitions in theory and practice: managing metals in the circular economy. Resources.

[bib41] Jin M. (2014). Advance of Cyclical Chemistry. Chem. World.

[bib42] Jones P.T., Geysen D., Tielemans Y., Van Passel S., Pontikes Y., Blanpain B., Quaghebeur M., Hoekstra N. (2013). Enhanced Landfill Mining in view of multiple resource recovery: a critical review. J. Clean. Prod..

[bib43] Klimek P., Obersteiner M., Thurner S. (2015). Systemic trade risk of critical resources. Sci. Adv..

[bib44] Klinglmair M., Fellner J. (2010). Urban mining in times of raw material shortage. J. Ind. Eco..

[bib45] Köhler A.R. (2013). Challenges for eco-design of emerging technologies: the case of electronic textiles. Mater.Des..

[bib46] Komeijani M., Ryen E.G., Babbitt C.W. (2016). Bridging the gap between eco-design and the human thinking system. Challenges.

[bib47] Krook J., Baas L. (2013). Getting serious about mining the technosphere: a review of recent landfill mining and urban mining research. J. Clean. Prod..

[bib48] Lang J.C. (2005). Zero landfill, zero waste: the greening of industry in Singapore. Int. J. Environ. Sustain. Dev..

[bib49] Lehmann M., de Leeuw B., Fehr E. (2014). Circular Economy: Improving the Management of Natural Resources.

[bib50] Li J., Zeng X., Chen M., Ogunseitan O.A., Stevels A. (2015). “Control-Alt-Delete”: rebooting solutions for the E-waste problem. Environ. Sci. Tech..

[bib51] Li J., Zeng X., Stevels A. (2015). Ecodesign in consumer electronics: past, present and future. Cri. Rev. Environ. Sci. Tech..

[bib52] Li J., Zhuo Y. (2019). Pushing Sustainable Development by Zero Waste Philosophy.

[bib53] Lifset R. (2014). Speaking industrial ecology. J. Ind. Eco..

[bib54] Linder M., Sarasini S., van Loon P. (2017). A metric for quantifying product-level circularity. J. Ind. Eco..

[bib55] Linton J., Yeomans J.S. (2004). Materials recycling and industrial ecology. Nat. Mater..

[bib56] Liu J., Hull V., Godfray H.C.J., Tilman D., Gleick P., Hoff H., Pahl-Wostl C., Xu Z., Chung M.G., Sun J. (2018). Nexus approaches to global sustainable development. Nat. Sustain..

[bib57] Liu J., Mooney H., Hull V., Davis S.J., Gaskell J., Hertel T., Lubchenco J., Seto K.C., Gleick P., Kremen C. (2015). Systems integration for global sustainability. Science.

[bib58] Liu J., Yu S., Shang Y. (2020). Toward separation at source: evolution of municipal solid waste management in China. Front. Environ. Sci. Eng..

[bib59] Liu Z., Adams M., Cote R.P., Chen Q., Wu R., Wen Z., Liu W., Dong L. (2018). How does circular economy respond to greenhouse gas emissions reduction: an analysis of Chinese plastic recycling industries. Renew. Sust. Energ. Rev..

[bib60] Liwarska-Bizukojc E., Bizukojc M., Marcinkowski A., Doniec A. (2009). The conceptual model of an eco-industrial park based upon ecological relationships. J. Clean. Prod..

[bib61] Lusty P.A.J., Gunn A.G. (2015). Challenges to global mineral resource security and options for future supply. Geol. Soc. Lond. Spec. Publ..

[bib62] Manhart A., Schleicher T., Degreif S. (2014). Global Circular Economy of Strategic Metals-The Best-Of-Two-Worlds Approach (Bo2W).

[bib63] Matete N., Trois C. (2008). Towards Zero Waste in emerging countries – a South African experience. Waste Manage.

[bib64] McLellan B., Yamasue E., Tezuka T., Corder G., Golev A., Giurco D. (2016). Critical minerals and energy–impacts and limitations of moving to unconventional resources. Resources.

[bib65] Meinert L., Robinson G., Nassar N. (2016). Mineral resources: reserves, peak production and the future. Resources.

[bib66] Mishra N., Kumar V., Chan F.T.S. (2011). A multi-agent architecture for reverse logistics in a green supply chain. Int. J. Prod. Res..

[bib67] Moreau V., Dos Reis P.C., Vuille F. (2019). Enough metals? Resource constraints to supply a fully renewable energy system. Resources.

[bib68] Mulvihill M.J., Beach E.S., Zimmerman J.B., Anastas P.T. (2011). Green chemistry and green engineering: a framework for sustainable technology development. Annu. Rev. Environ. Resour..

[bib69] Naeem S., Hahn D.R., Schuurman G. (2000). Producer-decomposer co-dependency influences biodiversity effects. Nature.

[bib70] O’Connor M.P., Zimmerman J.B., Anastas P.T., Plata D.L. (2016). A strategy for material supply chain sustainability: enabling a circular economy in the electronics industry through green engineering. ACS Sustain.Chem. Eng..

[bib71] O’Rourke D. (2014). The science of sustainable supply chains. Science.

[bib72] OECD (2019). Global Material Resources Outlook to 2060.

[bib73] Ogunseitan O.A., Schoenung J.M., Saphores J.D., Shapiro A.A. (2009). The electronics revolution: from e-wonderland to e-wasteland. Science.

[bib74] Ohno H., Matsubae K., Nakajima K., Kondo Y., Nakamura S., Fukushima Y., Nagasaka T. (2017). Optimal recycling of steel scrap and alloying elements: input-output based linear programming method with its application to end-of-life vehicles in Japan. Environ. Sci. Tech..

[bib75] Palmer P. (2005). Getting to Zero Waste.

[bib76] Plane J.M.C., Feng W., Dawkins E.C.M. (2015). The mesosphere and metals: chemistry and changes. Chemi. Rev..

[bib77] Poliakoff M., Licence P. (2007). Sustainable technology: green chemistry. Nature.

[bib78] Rathoure A.K. (2019). Zero Waste: Management Practices for Environmental Sustainability.

[bib79] Rauch J.N., Graedel T.E. (2007). Earth's anthrobiogeochemical copper cycle. Glob. Biogeochem Cycles.

[bib80] Reck B.K., Graedel T.E. (2012). Challenges in metal recycling. Science.

[bib81] Reuter M.A., Schaik A.v., Gutzmer J., Bartie N., Abadías-Llamas A. (2019). Challenges of the circular economy: a material, metallurgical, and product design perspective. Annu. Rev. Mater. Sci..

[bib82] Ruan J., Xu Z. (2016). Constructing environment-friendly return road of metals from e-waste: combination of physical separation technologies. Renew.Sust. Energ. Rev..

[bib83] Rustad J.R. (2012). Peak nothing: recent trends in mineral resource production. Enviro. Sci. Tech..

[bib84] Saidani M., Kendall A., Yannou B., Leroy Y., Cluzel F. (2019). Closing the loop on platinum from catalytic converters: contributions from material flow analysis and circularity indicators. J. Ind. Eco..

[bib85] Savaskan R.C., Bhattacharya S., Van Wassenhove L.N. (2004). Closed-loop supply chain models with product remanufacturing. Manage. Sci..

[bib86] Sheldon R.A. (2016). Green chemistry and resource efficiency: towards a green economy. Green. Chem..

[bib87] Song J., Han B. (2015). Green chemistry: a tool for the sustainable development of the chemical industry. Natl. Sci. Rev..

[bib88] Sun Z., Xiao Y., Agterhuis H., Sietsma J., Yang Y. (2016). Recycling of metals from urban mines – a strategic evaluation. J. Clean. Prod..

[bib89] Sverdrup H.U., Ragnarsdóttir K.V. (2014). Natural resources in a planetary perspective. Geochem. Perspect..

[bib90] Tercero Espinoza L., Schrijvers D., Chen W.-Q., Dewulf J., Eggert R., Goddin J., Habib K., Hagelüken C., Hurd A.J., Kleijn R. (2020). Greater circularity leads to lower criticality, and other links between criticality and the circular economy. Resour. Conserv. Recycl..

[bib91] The-Ellen-MacArthur-Foundation (2012). Towards the Circular Economy Vol. 1: An Economic and Business Rationale for an Accelerated Transition.

[bib92] Tilton J.E. (2015). World Metal Demand: Trends and Prospects.

[bib93] Trost B.M. (1995). Atom economy—a challenge for organic synthesis: homogeneous catalysis leads the way. Angew. Chem. Int. Ed..

[bib94] UNGA (2015). Transforming Our World: The 2030 Agenda for Sustainable Development.

[bib95] van Hullebusch E.D., Fontana D., Akcil A., Deveci H., Batinic B., Leal J.P., Gasche T.A., Ali Kucuker M., Kuchta K., Neto I.F.F. (2019). Recent advances on hydrometallurgical recovery of critical and precious elements from end of life electronic wastes - a review AU - Sethurajan. Manivannan. Cri. Rev. Environ. Sci.Tech..

[bib96] Vidal O., Goffe B., Arndt N. (2013). Metals for a low-carbon society. Nat. Geo..

[bib97] Wang Z., Huo J., Duan Y. (2020). The impact of government incentives and penalties on willingness to recycle plastic waste: an evolutionary game theory perspective. Front. Environ. Sci. Eng..

[bib98] Wang M., Tan Q., Chiang J.F., Li J. (2017). Recovery of rare and precious metals from urban mines—a review. Front. Environ. Sci. Eng..

[bib99] Wang Z., Zhang B., Guan D. (2016). Take responsibility for electronic-waste disposal. Nature.

[bib100] Waters C.N., Zalasiewicz J., Summerhayes C., Barnosky A.D., Poirier C., Gałuszka A., Cearreta A., Edgeworth M., Ellis E.C., Ellis M. (2016). The Anthropocene is functionally and stratigraphically distinct from the Holocene. Science.

[bib101] Wen Z., Zhang C., Ji X., Xue Y. (2015). Urban mining's potential to relieve China's coming resource crisis. J. Ind. Eco..

[bib102] Williams E. (2011). Environmental effects of information and communications technologies. Nature.

[bib103] Xu G., Yano J., Sakai S.-i. (2019). Recycling potentials of precious metals from end-of-life vehicle parts by selective dismantling. Environ. Sci. Tech..

[bib104] Ylä-Mella J., Pongrácz E. (2016). Drivers and constraints of critical materials recycling: the case of indium. Resources.

[bib105] Young C.-Y., Ni S.-P., Fan K.-S. (2009). Working towards a zero waste environment in taiwan. Waste Manage. Res..

[bib106] Zalasiewicz J., Williams M., Haywood A., Ellis M. (2011). The Anthropocene: a new epoch of geological time?. Philos. Trans. R. Soc. A.

[bib107] Zeng X., Ali S.H., Tian J., Li J. (2020). Mapping anthropogenic mineral generation in China and its implications for a circular economy. Nat. Commun..

[bib108] Zeng X., Li J. (2018). Urban mining and its resources adjustment: characteristics, sustainability, and extraction. Sci. Sin. Terrae.

[bib109] Zeng X., Li J., Shen B. (2015). Novel approach to recover cobalt and lithium from spent lithium-ion battery using oxalic acid. J. Hazard. Mat..

[bib110] Zeng X., Mathews J.A., Li J. (2018). Urban mining of E-waste is becoming more cost-effective than virgin mining. Environ. Sci. Technol..

