# Supplementary material for: Emerging anthropogenic circularity science: principles, practices, and challenges
Source: iScience. 2021 Feb 25;24(3):102237. doi: 10.1016/j.isci.2021.102237 (PMC7966983; doi:10.1016/j.isci.2021.102237)
Supplement: Document S1. Table S1 [file mmc1.pdf]

iScience, Volume *24*

Supplemental information

Emerging anthropogenic circularity science: principles, practices, and challenges

Xianlai Zeng and Jinhui Li

iScience, Volume *24*

Supplemental Information

Emerging Anthropogenic Circularity Science: Principles, Practices, and Challenges

Xianlai Zeng and Jinhui Li

Supplemental Table

Table S1 The collected data of global e-waste recycling

Country & region	Recycling rate (%)	Year	Country & region	Recycling rate (%)	Year
Australia	10	2008	Italy	32.1	2015
Austria	41	2016	Japan	30	2013
Belgium	34	2016	Latvia	23.2	2016
Brazil	10	2015	Liechtenstein	100	2016
Bulgaria	100	2016	Lithuania	38.9	2016
Canada	20	2014	Luxembourg	45.6	2016
China	46	2018	Macau	75	2014
Croatia	89.2	2016	Malta	11.5	2014
Cyprus	17	2014	Netherlands	40.4	2016
Czech Republic	46.1	2016	Nigeria	5	2012
Denmark	41.4	2016	Norway	49.3	2016
Egypt	10	2011	Poland	38.9	2016
Estonia	75.3	2016	Portugal	45.8	2016
Finland	42.1	2016	Romania	21.3	2014
France	37.1	2016	Slovakia	50.3	2016
Germany	39	2016	Slovenia	47.7	2015
Ghana	5	2012	Spain	37.4	2016
Greece	34.2	2016	Sweden	55.4	2016
Hungary	53.4	2016	Switzerland	73	2017
Iceland	41.3	2016	Taiwan	50	2011
India	5	2015	United Kingdom	49.8	2016
Ireland	49.5	2016	U.S.	43.9	2017

Note: data source from the refs. (ABS, 2013; Awasthi and Li, 2017; CEDARE, 2011; EDITORIALS, 2015; EUROSTAT, 2018; Islam et al., 2018; Kumar and Holuszko, 2016; Li et al., 2015; Song et al., 2014; USEPA, 2012; Zeng et al., 2017; Zeng et al., 2018);

Supplemental References

ABS (2013). ELECTRONIC AND ELECTRICAL WASTE. http://www.abs.gov.au/ausstats/abs@.nsf/Products/4602.0.55.005~2013~Main+Features~Electronic+and+Electrical+Waste?OpenDocument

Awasthi, A.K., and Li, J.H. (2017). Management of electrical and electronic waste: A comparative evaluation of China and India. Renewable & Sustainable Energy Reviews *76*, 434-447.

CEDARE (2011). Needs Assessment of the E-Waste Sector in Egypt.

EDITORIALS (2015). E-waste recycling still falling short (The Japan Times).

EUROSTAT (2018). Recycling rate of e-waste (Eurostat dissemination database).

Islam, M.T., Dias, P., and Huda, N. (2018). Comparison of E-Waste Management in Switzerland and in Australia: A Qualitative Content Analysis. International Journal of Environmental and Ecological Engineering *12*, 610-616.

Kumar, A., and Holuszko, M. (2016). Electronic Waste and Existing Processing Routes: A Canadian Perspective. Resources *5*, 35.

Li, J., Zeng, X., Chen, M., Ogunseitan, O.A., and Stevels, A. (2015). "Control-Alt-Delete": Rebooting Solutions for the E-waste Problem. Environmental Science & Technology *49*, 7095-7108.

Song, Q., Wang, Z., and Li, J. (2014). E-waste Management and Assessment in Macau (Deutschland, Germany: LAP LAMBERT Academic Publishing).

USEPA (2012). Recycling and waste electrical and electronic equipment management in Taiwan: A case study (The U.S. Environmental Protection Agency), pp. 32.

Zeng, X., Duan, H., Wang, F., and Li, J. (2017). Examining environmental management of e-waste: China's experience and lessons. Renewable and Sustainable Energy Reviews *72*, 1076-1082.

Zeng, X., Mathews, J.A., and Li, J. (2018). Urban Mining of E-Waste is Becoming More Cost-Effective Than Virgin Mining. Environmental Science & Technology *52*, 4835-4841.
